# New Biodegradable Polyester–Polyurethane Biocompositions Enriched by Urea

**DOI:** 10.3390/ma18163842

**Published:** 2025-08-15

**Authors:** Iwona Zarzyka, Beata Krzykowska, Karol Hęclik, Wiesław Frącz, Grzegorz Janowski, Łukasz Bąk, Tomasz Klepka, Jarosław Bieniaś, Monika Ostapiuk, Aneta Tor-Świątek, Magda Droździel-Jurkiewicz, Anita Białkowska, Adam Tomczyk, Anna Falkowska, Michał Kuciej

**Affiliations:** 1 Department of Organic Chemistry, Faculty of Chemistry, Rzeszów University of Technology, Powstańców Warszawy 6, 35-959 Rzeszów, Poland; b.krzykowska@prz.edu.pl; 2 Department of Biotechnology and Bioinformatic, Rzeszów University of Technology, Powstańców Warszawy 6, 35-959 Rzeszów, Poland; kheclik@prz.edu.pl; 3 Department of Material Forming and Processing, The Faculty of Mechanical Engineering and Aeronautics, Rzeszów University of Technology, Powstańców Warszawy 12, 35-959 Rzeszów, Poland; wf@prz.edu.pl (W.F.); gjan@prz.edu.pl (G.J.); lbak@prz.edu.pl (Ł.B.); 4 Department of Technology and Polymer Processing, Faculty Mechanical Engineering, Lublin University of Technology, Nadbystrzycka 36, 20-618 Lublin, Poland; t.klepka@pollub.pl (T.K.); a.tor@pollub.pl (A.T.-Ś.); 5 Department of Materials Engineering, Faculty Mechanical Engineering, Lublin University of Technology, Nadbystrzycka 36, 20-618 Lublin, Poland; j.bienias@pollub.pl (J.B.); m.ostapiuk@pollub.pl (M.O.); m.drozdziel@pollub.pl (M.D.-J.); 6 Faculty of Mechanic, Radom University, Stasieckiego 54, 26-600 Radom, Poland; a.bialkowska@uthrad.pl; 7 Department of Mechanics and Applied Computer Science, Faculty of Mechanical Engineering, Bialystok University of Technology, 45C Wiejska St, 15-351 Bialystok, Poland; a.tomczyk@pb.edu.pl (A.T.); a.falkowska@pb.edu.pl (A.F.); m.kuciej@pb.edu.pl (M.K.)

**Keywords:** natural polyester, polyurethane, urea, morphology, molecular interactions, biodegradability, mechanical properties

## Abstract

Novel polyester–polyurethane polymeric materials were formulated by combining a natural aliphatic polyester, poly(3-hydroxybutyrate) (P3HB), with a synthetic aliphatic polyurethane via melt blending. The resulting fully biodegradable compositions were functionally modified through the incorporation of urea, with the aim of enabling post-consumer utilization of the material residues as nitrogen-rich fertilizers. The fabrication process was systematically established and optimized, focusing on homogeneous blending and processability. Comprehensive mechanical characterization—including tensile strength, impact resistance, and Shore hardness—was performed. Among the tested formulations, composites containing 1 wt.% urea demonstrated superior mechanical performance and optimal processing behavior. Fourier-transform infrared (FTIR) spectroscopy was employed to investigate molecular-level interactions between polymeric phases and urea, while scanning electron microscopy (SEM) was utilized to assess the morphological characteristics of the resulting biocompositions. Comparative analyses of the physico-mechanical properties and biodegradability were conducted among the urea-modified compositions, binary P3HB–polyurethane blends, and neat P3HB. The observed improvements in mechanical integrity and functional biodegradability suggest that the developed urea-enriched compositions are promising candidates for the fabrication of eco-friendly seedling pots via injection molding technology.

## 1. Introduction

The accumulation of persistent plastic waste in terrestrial and aquatic ecosystems has raised critical environmental and public health concerns, prompting the development of sustainable and biodegradable polymer alternatives [1,2]. Among various candidates, poly(3-hydroxybutyrate) (P3HB), a microbial aliphatic polyester belonging to the family of polyhydroxyalkanoates (PHAs), has attracted considerable interest due to its biodegradability, biocompatibility, and biosynthetic origin [3,4,5]. Despite its ecological advantages, the commercial use of P3HB is hampered by its brittleness, narrow processing window, and low impact resistance [6,7].

One widely used approach to overcoming these limitations is the blending of P3HB with other polymers to improve its mechanical and thermal properties. Synthetic aliphatic polyurethanes are attractive blending partners owing to their flexibility, durability, and tunable mechanical properties [8,9]. The incorporation of polyurethanes into biopolyester matrices has been shown to improve elasticity and impact strength while maintaining partial biodegradability, especially when aliphatic diisocyanates are used [10,11].

In addition to improving performance characteristics, the design of modern biodegradable materials increasingly integrates post-use functionality to align with circular economy principles. One promising strategy involves the enrichment of polymer matrices with agrochemicals such as urea, enabling the polymer residues to serve as slow-release nitrogen fertilizers after degradation [12,13]. Urea, a commonly used nitrogen source in agriculture, can be physically embedded or chemically bonded within polymer systems to modulate its release profile and reduce nutrient leaching [14,15].

Furthermore, the potential agricultural application of such multifunctional materials—such as biodegradable seedling pots—has garnered increasing attention. These applications require materials that not only degrade in soil conditions but also possess sufficient mechanical strength during handling and transplantation [16,17].

Biodegradable polyester–polyurethane (PU) blends represent an attractive alternative to conventional plastics, combining biodegradability and biocompatibility with the ability to tailor mechanical and processing properties [10,18,19,20,21]. In particular, the incorporation of natural or synthetic aliphatic polyesters such P3HB, poly(ε-caprolactone) (PCL), or polylactic acid (PLA) into PU matrices allows the design of materials with controlled degradation behavior under biological or environmental conditions [22,23]. Studies have shown that the biodegradability of such blends depends on several factors, including the type and content of polyester, degree of crystallinity, surface area accessible to microorganisms, and phase morphology [24,25,26].

For P3HB/PU blends, the presence of the stiff, crystalline P3HB phase has been found to facilitate the initiation of biodegradation by promoting water and enzyme penetration, especially when the material exhibits porous structures [27,28]. Meanwhile, the polyurethane segments, depending on their chemical composition (e.g., aliphatic vs. aromatic diisocyanates), can undergo enzymatic or hydrolytic degradation to varying extents [29,30]. Phase separation phenomena also play a crucial role; significant incompatibility between phases may lead to microphase separation, which can reduce mechanical properties but simultaneously promote selective degradation of the more hydrophilic phase [31].

Biodegradation behavior of PLA/PU and PCL/PU blends has also been extensively studied. PLA degrades relatively quickly under composting conditions; however, its brittleness and stiffness can be mitigated by incorporating PU as an elastomeric phase [20,32,33]. Conversely, PCL, due to its low melting point and flexibility, enhances the plasticity of the composite, and its slower degradation rate can be balanced by the presence of more hydrophobic or amorphous PU components [21,34]. Understanding the balance between in-use stability and biodegradability is key for tailoring these materials to specific applications, ranging from biomedical devices to disposable packaging [20,24,35].

In this study, novel biodegradable polymer composites based on P3HB and aliphatic polyurethane were developed and modified with low concentrations of urea to impart post-degradation fertilizing properties. The composites were synthesized via melt blending, and their mechanical, morphological, and chemical properties were thoroughly characterized using standard analytical techniques, including Fourier-transform infrared (FTIR) spectroscopy and scanning electron microscopy (SEM). The performance of the urea-modified systems was benchmarked against neat P3HB and unmodified P3HB–PU blends. The results highlight the potential of these materials for agricultural applications, particularly in the production of biodegradable injection-molded horticultural products.

## 2. Materials and Methods

### 2.1. Materials

Poly(3-hydroxybutyrate) (P3HB) was obtained from a commercial supplier (Biomer, Germany). An aliphatic polyurethane (PU) was synthesized using polyethylene glycol and hexamethylene diisocyanate (HDI) following established protocols [36]. Urea (analytical grade) was purchased from Sigma-Aldrich. All reagents were used as received without further purification.

### 2.2. Preparation of Polymer Compositions

The composites were prepared by melt blending in a twin-screw extruder (ZAMAK REA-2P12A Explorer). The blend ratios of P3HB to PU were fixed at 90:10 wt.% (P3HB–PU) based on preliminary optimization. Urea was added at concentrations of 1.0 and 2.0 wt.% (C1 and C2, respectively). The prepared composition was dried before the extrusion process at a temperature of approx. 30–35 °C for 24 h.

The extruder used in the study has a screw diameter of 12 mm and a working length of 480 mm, which corresponds to a relative length of L/D = 40, typical for laboratory devices intended for intensive mixing and reactive processing. The screw system consisted of plasticizing, transport, and grinding zones, which provided appropriate conditions for homogenization of the liquid and solid phases. The temperature was controlled in seven independent heating and cooling zones and on the head. The melt blending was carried out at the temperature range given in Table 1 with a screw speed of 130 rpm (Table 2). The extrudate was cooled, pelletized, and then injection-molded into standard test specimens.

### 2.3. Injection Molding of Shapes for Testing Mechanical Properties

A BOY 55E injection molding machine was used to produce samples for testing the physical and mechanical properties. The injection parameters are listed in Table 3. The injection mold temperature was 25 °C for P3HB and 30 °C for the tested compositions.

### 2.4. Methods

#### 2.4.1. Mechanical Tests

Mechanical tests were performed for at least 5 samples from each series. Static tensile test was performed according to EN ISO 527:2019 [37]. Due to the fact that the samples with 2% urea content were too brittle, no tensile tests were performed on them. Bending test was performed according to EN ISO 178:2019 [38]. Due to the excessive brittleness of the composites containing 2% urea, it was not possible to test them in bending; the samples broke prematurely, which prevented correct measurement. Hardness was determined by the ball indentation method according to PN-EN ISO 2039-1:2004 [39].

#### 2.4.2. Scanning Electron Microscopy

The analysis of the morphology of the polymer compositions was carried out using a Gemini 360 scanning electron microscope, operating at an accelerating voltage of 20 kV and using secondary electron detection (SEI). The samples were prepared by freezing them in liquid nitrogen and then mechanically crushing them using a hammer. The prepared samples were placed in the microscope chamber. Observations were carried out in variable vacuum mode, analyzing the surface structure in selected micro-areas. The materials were not sprayed with a conductive layer.

#### 2.4.3. Infrared Spectroscopy Measurements

Fourier-transform infrared (FTIR) spectra of P3HB, polyurethane, and their compositions were recorded on a Bruker ALPHA FTIR instrument in ATR mode for compositions or in KBr pellets for P3HB in the wavenumber range of 4000–450 cm^−1^ at a resolution of 2 cm^−1^.

#### 2.4.4. Biodegradability Test

A 12-week biodegradation process was carried out based on the EN 13432:2002 Standard [40]. Biodegradation was carried out in aerobic conditions in the composting process at a temperature of 50 °C. For each of the tested polymers, glass containers containing compost were prepared, the moisture content of the samples was selected, and 4 samples (20 × 20 × 2 mm) of the tested material were introduced. For each of the tested polymers, the biodegradation process was performed in duplicate. During the biodegradation process, the total dry mass loss was determined.

## 3. Results and Discussion

### 3.1. Formulation and Manufacturing of Biodegradable P3HB–PU–Urea Compositions

**Figure 1:**
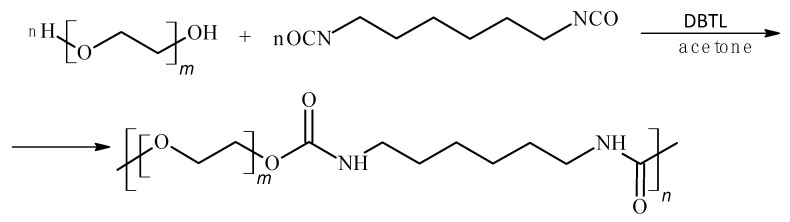
Reaction scheme of polyurethane synthesis. *m* = 9 and *n* = 23.

To develop an optimal material composition that combines structural integrity with high biodegradability and post-use fertilizing functionality, poly(3-hydroxybutyrate) (P3HB) was blended with an aliphatic thermoplastic polyurethane and urea. The polyurethane was synthesized from polyethylene glycol (PEG, Mn = 400 g/mol) and hexamethylene diisocyanate (HDI) in the presence of dibutyltin dilaurate (DBTL) as a catalyst following the reaction scheme in Figure 1.

The resulting polyurethane exhibited a number-average molecular weight (Mn) of 8523 g/mol, a weight-average molecular weight (Mw) of 13,229 g/mol, and a dispersity index (Đ) of 1.55.

The synthesized polyurethane was used to prepare composites containing 10 wt.% PU and either 1 or 2 wt.% urea, with the remainder consisting of P3HB. The components were homogenized and processed in the molten state using a twin-screw extruder. Stable processing conditions were established, enabling the production of materials with reproducible processing parameters and consistent performance characteristics.

### 3.2. Analysis of the Processing Conditions of P3HB–PU–Urea Blends

The processing conditions of polymer compositions based on P3HB, PU, and urea at different concentrations (1% and 2% wt.%) were analyzed to determine the effect of material composition on the extrusion process, with particular attention to the thermal parameters, pressure level, drive system load, and general process stability.

For the composition containing 10 wt.% PU without urea additive, the temperature profile was set to 152 °C in zones 3–7, 150 °C in zone 2, 145 °C in zone 1, and 152 °C at the die, with the feed zone maintained at 50 °C. This high temperature level indicates a relatively high melt viscosity of the material, which is also confirmed by the measured pressure (28 bar) and drive system load (83%). This suggests that such a composition, although processable, generates significant flow resistance and requires high energy input for plasticization and homogenization.

The addition of 1 wt.% urea allowed a reduction of the temperature profile along the entire length of the system to 150 °C (die and zones 3–7), 148 °C for zone 2, and 145 °C for zone 1, without deteriorating the course of the process. A significant drop in operating pressure (to 13 bar) and screw load (to 58%) was also recorded, which indicates the effective role of urea as a low-molecular-weight modifier of supramolecular structures. Its presence could have influenced the increased mobility of polymer chain segments, lowering the overall viscosity of the mixture and facilitating melt flow through the screw system. Lower mechanical load may also positively affect the operational durability of the equipment and reduce the risk of material overheating in high-shear zones.

In the case of using a higher urea content (2 wt.%), the temperature profile was further reduced, reaching values of 141–147 °C in the heating zones and 148 °C at the die. Despite the further temperature drop, the process remained stable, as evidenced by reduced operating pressure (10 bar) and drive load (51%). These parameters may indicate even greater fluidization of the composition; however, from the processing point of view, this may pose a potential risk of incomplete homogenization or insufficient dispersion of the composite components, especially when residence time in the processing zone is shortened. It is worth noting that very low-pressure values may limit the efficiency of the mechanical mixing of components, especially in systems containing volatile or easily sublimating substances.

The observed changes in the pressure and load parameters in response to varying urea contents clearly indicate its beneficial effect on the processability of the mixture in the context of extrusion. Particularly, the composition containing 1 wt.% urea appears to be the most optimal in terms of the balance between viscosity and mixing quality. On the other hand, the system with 2 wt.% urea, despite favorable energy parameters, requires further evaluation in terms of the homogeneity of the resulting composites and their physicochemical properties. The collected data can be used for further optimization of the extrusion process and serve as a starting point for scaling the technology from the laboratory to semi-industrial scale. In particular, it is important to verify the influence of processing parameters on the morphology of the obtained materials, as well as their mechanical properties.

### 3.3. FTIR Analysis of Material Structure and Intermolecular Interactions

**Figure 2:**
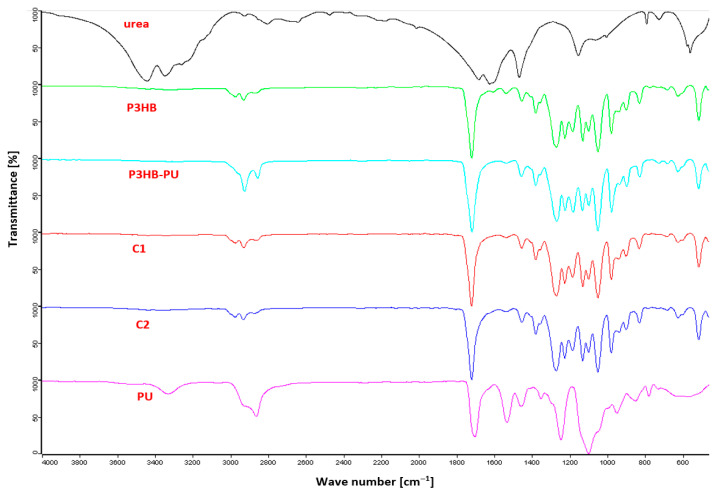
Set of FTIR spectra P3HB, PU, composition P3HB with 10 wt.% PU (P3HB–PU), urea, and compositions of P3HB with 10 wt.% PU and 1 or 2 wt.% urea (C1 and C2).

The structure of the materials and intermolecular interactions of the components were analyzed based on FTIR spectroscopy. Figure 2 shows a comparison of FTIR spectra of urea, P3HB, polyurethane, the P3HB–PU composition, and compositions with urea (C1 and C2).

In the FTIR spectrum of P3HB, absorption bands were observed at 2973.84 and 2929.95 cm^−1^, originating from stretching vibrations of C–H bonds, indicating the presence of CH_2_ and CH_3_ groups. At the wavenumber of 1718.31 cm^−1^, a characteristic band for ester carbonyl (C=O) stretching vibrations was detected. Bands in the range of 1268.21 and 1128.10 cm^−1^ correspond to asymmetric and symmetric stretching vibrations of C–O–(CO) bonds in the ester group. The band at 1097.47 cm^−1^ arises from deformation vibrations of secondary alcohol C–OH bonds. Characteristic FTIR absorption bands of P3HB include C–H stretching vibrations near 2973 and 2929 cm^−1^, ester C=O stretching at ~1718 cm^−1^, and C–O/C–OH stretches between 1268 and 1097 cm^−1^, consistent with previous reports [41,42].

The FTIR spectrum of polyurethane shows characteristic bands corresponding to typical functional groups of this polymer structure. An absorption band centered at 3330.91 cm^−1^ indicates the presence of N–H bonds. This band is quite broad due to hydrogen bonding between urethane groups within the polymer chains [43]. At 2862.26 cm^−1^, bands related to stretching vibrations of C–H groups originating from aliphatic CH_2_ chains were visible. A key band in the polyurethane spectrum is a strong absorption band near 1700.83 cm^−1^, corresponding to carbonyl (C=O) stretching vibrations in urethane groups. An absorption band at 1531.03 cm^−1^ corresponds to N–H bending vibrations. In the lower range, at 1247.07 cm^−1^, bands from C–N stretching vibrations were observed. Additionally, a band at 1097.24 cm^−1^ indicates the presence of C–O–C and C–OH vibrations characteristic of ether and primary alcohol groups, respectively. The broad absorption band at ~3330 cm^−1^ corresponds to N–H stretching vibrations involved in hydrogen bonding within urethane groups, in agreement with previous FTIR analyses of PU elastomers [44,45].

In the FTIR spectrum of the P3HB composite with 10 wt.% PU, a distinct absorption band for the C=O group was observed at 1718.56 cm^−1^, common for both ester groups of P3HB and urethane groups of PU. Stretching vibrations at 2924.95 and 2854.79 cm^−1^ were characteristic for C–H bonds in methyl and methylene groups. Absorption bands at 1266.72 and 1129.50 cm^−1^ corresponded to asymmetric and symmetric stretching vibrations of C–O–C and C–OH bonds [43,45].

The FTIR spectrum of urea revealed characteristic absorption bands for urea: N–H stretching vibrations at 3442.10 and 3347.63 cm^−1^, suggesting the presence of hydrogen bonding. A band at 1680.78 cm^−1^ corresponded to carbonyl (C=O) stretching vibrations. Absorption bands at 1622.96 and 1456.35 cm^−1^ were attributed to N–H bending vibrations, with the latter confirming the presence of hydrogen bonds C=O⋯HN. At 1152.30 cm^−1^, deformation bending vibrations involving C–N bonds characteristic for amide structures were present. The band at 723.25 cm^−1^ indicated twisting deformation vibrations of NH_2_ groups [45,46].

**Figure 3:**
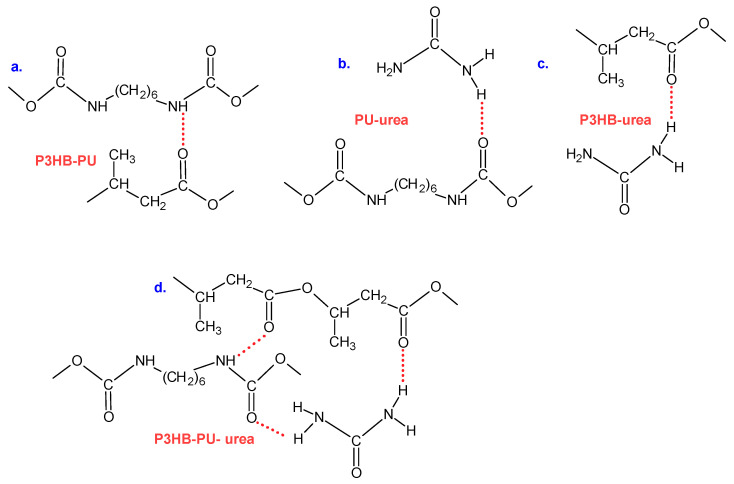
Scheme illustrating possible intermolecular hydrogen bonds in the polyester–polyurethane–urea composition: (**a**) between P3HB and PU, (**b**) between PU and urea, (**c**) between P3HB and urea, and (**d**) between P3HB and urea and PU.

In the FTIR spectrum of the P3HB–PU composite enriched with 1 wt.% urea, an absorption band in the range of wavenumbers 3650–3100 cm^−1^ was registered, corresponding to N–H stretching vibrations. This band was broad and diffuse, confirming the existence of intermolecular hydrogen bonds between urea and polyester, as well as between urea and polyurethane (Figure 3). The introduction of low-molecular-weight urea, which is more mobile and has a large number of hydrogen atoms, clearly favors the formation of hydrogen bonds, which is not observed in the spectrum of the P3HB–PU blend alone. The amount of urea added is not large; therefore, the intensity of this band is low. However, this band appears and increases in intensity with increasing the urea content in the P3HB–PU blend.

Absorption bands at 2973.31 and 2929.40 cm^−1^ indicated the presence of stretching vibrations of C–H bonds in CH_3_ and CH_2_ groups. The most intense band at 1719.19 cm^−1^ corresponded to stretching vibrations of C=O bonds, indicating the presence of carbonyl groups from esters, urethanes, and urea. The absorption band at 1534.55 cm^−1^ indicated N–H bending vibrations of urethane and urea groups. A band at 1377.91 cm^−1^ was attributed to deformation vibrations of –NH_2_ groups. Bands at 1097.68 and 1048.26 cm^−1^ corresponded to stretching vibrations of C–O–C and C–N bonds, also characteristic for C–OH groups [45,46]. The broadening and shifting of N–H and C=O absorption bands in P3HB–PU–urea composites indicate intermolecular hydrogen bonding, consistent with previous composite FTIR studies [44].

The FTIR spectrum of the P3HB–PU composite enriched with 2 wt.% urea showed analogous bands to the 1 wt.% urea sample. The N–H stretching bands at 3442 and 3348 cm^−1^, indicating the presence of N–H bonds, were especially pronounced in the urea sample. In the spectra of P3HB and the 1 wt.% urea composite, this band was significantly weaker and broader, which may indicate lower amounts of these groups or their involvement in hydrogen bonding. In pure PU, this band was observed at 3331 cm^−1^, typical for urethane N–H groups. The carbonyl band best represents the spectra of P3HB and pure polyurethane at 1718.31 cm^−1^ and 1700.83 cm^−1^, respectively. In the urea sample, this band was weaker and shifted toward lower wavenumbers due to the amide structure [42,44,45].

### 3.4. Effect of Urea and Aliphatic Polyurethane Additives on the Fracture Surface Morphology of Poly(3-hydroxybutyrate) Compositions

**Figure 4:**
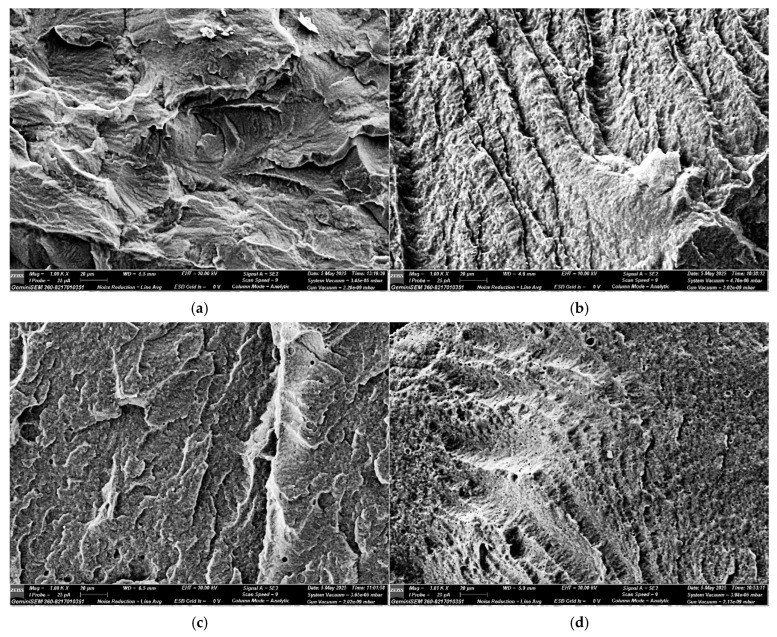
SEM micrographs: (**a**) P3HB, (**b**) P3HB–PU compositions, and (**c**,**d**) compositions enriched with 1 and 2 wt.% urea C1 and C2, respectively.

Figure 4a,b present SEM micrographs of the fracture surfaces of poly(3-hydroxybutyrate) (P3HB) without modifiers (Figure 4a) and of the matrix modified with aliphatic polyurethane (Figure 4b). This modifier was synthesized from HDI and short-chain polyethylene glycol with a molecular weight of 400 g/mol. The thus-formed polymer matrix was further modified with varying amounts of a low-molecular-weight modifier—urea at 1 wt.% (Figure 4c) and 2 wt.% (Figure 4d).

The fracture images of the damaged samples allow preliminary explanation of the mechanisms and effectiveness of the applied modifier. All micrographs were obtained by scanning the fracture surfaces of the samples at the crack initiation sites under applied loads.

In Figure 4a, the fracture surface of neat P3HB is distinctly crystalline and glassy, confirming the high degree of crystallinity of P3HB and suggesting a regular crack propagation pathway [47].

The polyreaction of P3HB with aliphatic polyurethane already causes a visible change in the fracture surface (Figure 4b). Small crystalline regions of P3HB oriented in various directions become less visible, while the edges of the ordered P3HB domains become smoother in the matrix and transform into wavy, unidirectionally aligned rough domains, indicating plasticization of the original biopolymer by the short-chain aliphatic PU [47,48].

The introduction of 1 wt.% urea results in uniform distribution of urea in the sample, which is only noticeable in Figure 4c presenting the composite containing 1 wt.% urea. In Figure 4c, clear separations typical of the produced matrix glassy fractures (compare Figure 4a and Figure 4b) are visible, separated by PU-derived wavy regions and only sporadically (compared to Figure 4c) identified crystalline substance attributed to urea. This may suggest tertiary interactions between the matrix and urea, which may potentially positively influence the mechanical properties of the produced biomaterials [49].

The addition of 2 wt.% urea (Figure 4d) results in unevenly distributed and unidirectionally aligned aforementioned rough regions. Clusters of crystalline substance are noticeable, indicating probable urea agglomeration in the sample, which is expected to deteriorate the mechanical properties of the biopolymer composition [50].

### 3.5. Mechanical Properties of the Polymer–Urea Biocompositions

**Figure 5:**
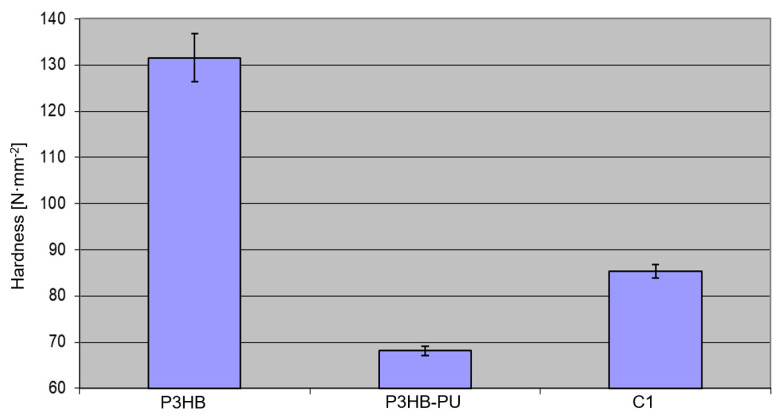
Hardness of the tested materials: P3HB and its compositions with 10 wt.% PU (P3HB–PU) and with 10 wt.% PU and 1 wt.% urea (C1).

Based on the presented data (Figure 5) regarding the hardness of polymeric biomaterials, a detailed comparative analysis was carried out for the tested materials: neat P3HB, the P3HB–PU composition, and the P3HB–PU composition with an additional 1 wt.% urea (C1). The analysis was based on mean Brinell hardness values (HB) and corresponding standard deviations.

Neat P3HB exhibited the highest hardness of approximately 130 N/mm^2^, with a relatively high standard deviation of 5.25 N/mm^2^. This indicates that P3HB is characterized by considerable stiffness and resistance to surface deformation, which may result from its highly crystalline structure and absence of a soft segment phase. The relatively large deviation may be attributed to microstructural heterogeneity, such as local crystalline orientation or processing irregularities [51].

Modification of P3HB with 10 wt.% PU led to a significant decrease in hardness to ~68 N/mm^2^, accompanied by a substantial reduction in standard deviation to 0.99 N/mm^2^. This suggests that the PU addition notably softens the material by introducing flexible urethane segments into the structure, reducing brittleness and stiffness while improving the uniformity of mechanical performance (lower variability). This effect may be desirable in applications requiring enhanced flexibility or fracture resistance [52].

The addition of 1 wt.% urea to the P3HB–PU composition resulted in an increase in hardness to approximately 85 N/mm^2^, with a slightly higher, but still low, standard deviation of 1.45 N/mm^2^. This increase indicates that urea may act as a crosslinking agent by forming additional hydrogen bonds between the polyurethane segments and the P3HB matrix, thus increasing the stiffness of the material without significantly compromising the uniformity of its mechanical properties. It is also possible that urea plays a role as a crystallization-promoting additive, enhancing the secondary ordering of polymer chains.

Tensile testing revealed an increase in elongation at break for P3HB samples containing 10 wt.% PU compared to neat P3HB. However, the addition of 1 wt.% urea to the composition caused a notable reduction in this parameter—by approximately 80%—bringing the elongation at break close to that of neat P3HB. At the same time, the tensile strength decreased by around 20% compared to neat P3HB and was also approximately 5% lower than the tensile strength of the P3HB–PU composition [53].

The incorporation of 10 wt.% PU into P3HB (P3HB–PU) caused a decrease in Young’s modulus. Conversely, the addition of 1 wt.% urea to this composition (C1) significantly increased Young’s modulus—by as much as 80%—compared to neat P3HB.

**Figure 6:**
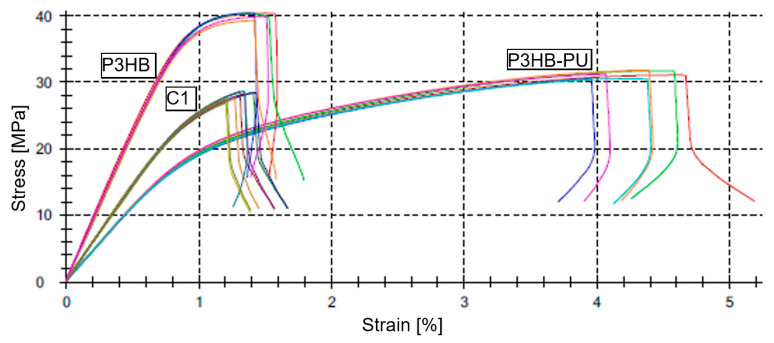
Stress–strain curves obtained from uniaxial tensile tests for the materials: neat P3HB and its compositions with 10 wt.% PU (P3HB–PU) and with 10 wt.% PU and 1 wt.% urea (C1).

The introduction of 2 wt.% urea prevented the fabrication of test specimens. Samples cracked while still in the mold cavity. Upon mold opening, the brittle specimen disintegrated into small fragments under the action of the ejection system. The test results are summarized in Figure 6 and Table 4.

**Figure 7:**
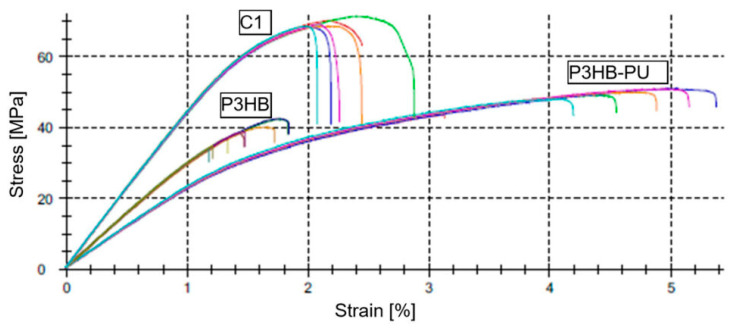
Representative stress–strain characteristics obtained from the three-point bending test for the analyzed materials: neat P3HB and its compositions with 10 wt.% PU (P3HB–PU) and with 10 wt.% PU and 1 wt.% urea (C1).

Flexural strength tests were carried out in accordance with DIN EN ISO 178, and the analysis of mechanical parameters is presented in Table 5 and Figure 7.

Neat P3HB polyester exhibited the highest flexural modulus, approximately 4513 MPa. The high stiffness of this material results from its crystalline structure and the absence of additives that could loosen the polymer chains. The incorporation of PU significantly reduced the flexural modulus to about 2321 MPa, which can be directly attributed to the elastic nature of PU. PU introduces a softer phase into the composite system due to its segmented structure (hard and soft segments), thus imparting a plasticizing effect and enhancing deformability at the expense of elastic deformation resistance.

An interesting observation is the increase in flexural modulus to approximately 3054 MPa upon the addition of 1 wt.% urea to the P3HB–PU composition. This may be due to the presence of polar functional groups (–NH_2_ and –C=O) in the urea structure, which can engage in hydrogen bonding interactions with both PU segments and the carbonyl groups of P3HB, resulting in partial stiffening of the material structure. This effect may also stem from enhanced crosslinking density or reduced chain mobility, which increases the resistance of the material to elastic deformation [54].

The flexural strength (σ_fM_) and stress at break (σ_fB_) values for neat P3HB were approximately 69 MPa, indicating its brittle nature; the material fractured immediately after reaching its load-bearing limit without transitioning into a plastic deformation phase. This failure mechanism is typical for highly crystalline polyesters, where the absence of mobile segments leads to stress concentration and crack initiation without prior plasticity.

In contrast, the P3HB–PU composite showed σ_fM_ and σ_fB_ values of around 50 MPa. Although this represents a significant reduction compared to the base material, it was accompanied by a notable increase in deformability, indicating a shift in failure behavior from brittle to ductile. PU likely acts as a stress-dissipating phase, enabling a more uniform stress distribution and delaying crack initiation.

However, the addition of 1 wt.% urea (C1) noticeably deteriorated the flexural strength, reducing the σ_fM_ and σ_fB_ to about 39 MPa. This suggests that the presence of urea compromises the structural integrity of the material. Possible mechanisms include insufficient compatibility between urea and the polymer phase, local segregation of the additive leading to microdefects, or potential crystallization of urea within the matrix, which could act as crack initiation sites [55].

The flexural strain at maximum load (ε_fM_) and at break (ε_fB_) values are consistent with the previously described plasticity characteristics (Figure 7). For neat P3HB, these values were as low as 2.14%, indicating a very limited ability to absorb energy before failure. The incorporation of PU increased these values to approximately 4.62% (P3HB–PU), confirming the beneficial effect of the modification in enhancing material ductility and resistance to crack propagation.

In contrast, the inclusion of 1 wt.% urea (C1) significantly reduced deformability; ε_fM_ and ε_fB_ decreased to 1.47%, supporting the hypothesis of a degrading influence of this additive on the material structure. Such deterioration in mechanical behavior may also result from heterogeneous dispersion of urea and disrupted interfacial adhesion at the P3HB–PU boundary, which leads to premature crack initiation and reduced energy absorption capacity.

### 3.6. Biodegradability Assessment of Biocomposites

Biodegradation of the tested materials was carried out under aerobic conditions, through composting at a temperature of 50 °C. The analysis of dry mass loss of the samples during the 90-day biodegradation process was performed by comparison with control samples that were not subjected to degradation.

**Figure 8:**
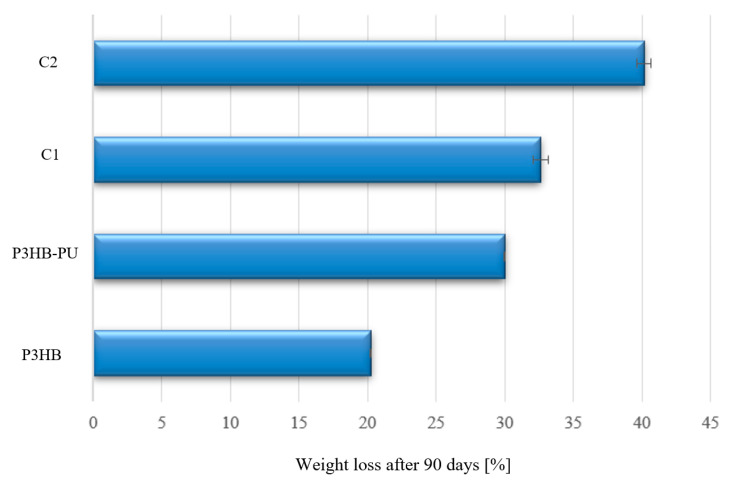
Percentage of mass loss of samples subjected to biodegradation after 90 days, i.e., P3HB and its biocompositions with 10 wt.% of PU (P3HB–PU) and 1 or 2 wt.% of urea (C1 and C2, respectively).

The graph in Figure 8 shows that the greatest mass loss, approximately 40 wt.%, occurred in the case of the composition enriched with 2 wt.% of urea, labeled C2. A similar degree of mass loss was observed for the C1 and P3HB–PU composites. The lowest dry mass loss was observed for the P3HB sample at the level of 20 wt.%. These results confirm that the presence of polyurethane does not inhibit the biodegradation of P3HB but rather accelerates it, which is associated with the presence of hydrophilic groups increasing water retention in the material, thus facilitating the biodegradation process. The addition of urea further enhances the degradation rate [56,57].

**Figure 9:**
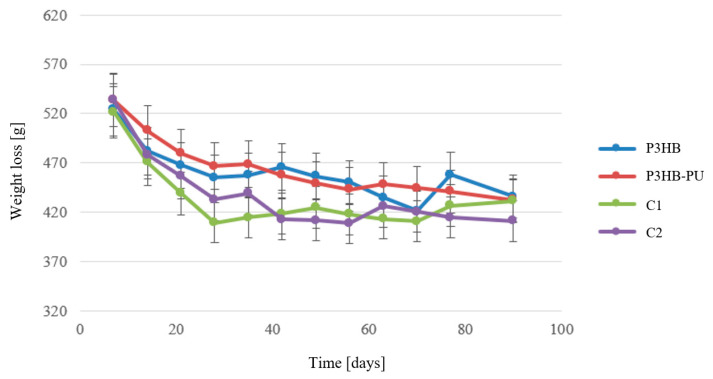
Mass loss of samples during the biodegradation process of P3HB and its biocompositions with 10 wt.% of PU (P3HB–PU) and 1 or 2 wt.% of urea (C1 and C2, respectively).

Analyzing the mass loss over time (Figure 9), it was observed that all samples exhibited the highest mass losses during the first 30 days of the biodegradation process. This indicates more intensive microbial metabolic activity and indirectly points to a high degradation rate of the materials, as shown in Figure 9.

It is noteworthy that the greatest mass losses were observed in samples containing urea. In the next stage—during the second month of the biodegradation process—a clear slowdown in degradation was observed for all samples. The smallest changes in mass were noted for the samples without urea (P3HB and P3HB–PU) [56].

## 4. Conclusions

The processing of P3HB-based composites with the addition of 10 wt.% polyurethane (PU) and urea showed good moldability for urea contents up to 1 wt.%. At a higher urea concentration (2 wt.%), significant technological issues were observed; molded parts cracked already in the forming cavity, indicating a deterioration of processing properties and structural integrity.

FTIR spectroscopy confirmed the presence of characteristic functional groups for P3HB and PU and indicated potential hydrogen bonding interactions between carbonyl groups of P3HB, urethane segments of PU, and –NH_2_ groups from urea. These signals suggest physicochemical interactions between the components rather than a purely physical mixture.

SEM imaging revealed a distinct change in surface morphology, from smooth and homogeneous for pure P3HB to rougher and heterogeneous in composites containing PU and urea. Particularly in samples with urea, the presence of fine inclusions and microcracks was observed, which may be attributed to limited compatibility between urea and the polymer matrix.

Material hardness (HB method) decreased significantly after the PU addition (from approx. 130 to 68 N/mm^2^), indicating a softening effect. The addition of 1 wt.% urea increased hardness to approx. 85 N/mm^2^, suggesting partial crosslinking through hydrogen bonds that locally stiffen the structure.

Tensile testing showed that the P3HB–PU composite significantly increased elongation at break compared to neat P3HB, though accompanied by a reduction in tensile strength. The addition of 1 wt.% urea (C1) reduced elongation to a level similar to pure P3HB but increased Young’s modulus by up to 80%, confirming structural stiffening.

In bending tests, neat P3HB exhibited the highest flexural modulus (~4500 MPa), confirming its stiff and brittle character. The addition of PU lowered the modulus to approx. 2320 MPa and transitioned the material to a more ductile behavior. Urea addition partially compensated for the softening effect, increasing the modulus to approx. 3050 MPa, but at the expense of lower strength and deformability at break.

Biodegradation under composting conditions (50 °C, 90 days) showed that all modifications accelerated the degradation of P3HB. The addition of PU and, particularly, urea (1–2 wt.%) intensified the mass loss (up to 40%), likely due to increased hydrophilicity and greater accessibility for microorganisms. The most dynamic degradation occurred within the first 30 days for all samples.

The use of small amounts of urea (1 wt.%) may act as both a crosslinking agent and a biodegradation promoter; however, its content must be precisely controlled. Excess urea deteriorates mechanical performance, disrupts microstructural homogeneity, and impairs processing.

Application potential: The developed P3HB-based biocomposites modified with polyurethane and low urea content show promise for biodegradable applications requiring a balance of flexibility, reduced brittleness, and controlled stiffness. Such materials could be suited for short life cycle products in agriculture as films, seedling containers, and nails for agrotextiles, where mechanical tunability and environmental degradability are simultaneously required.

## Figures and Tables

**Table 1:** Temperature profile in individual heating zones of the screw extruder.

Material	Head	Zone 7	Zone 6	Zone 5	Zone 4	Zone 3	Zone 2	Zone 1	Feed
P3HB	158	152	152	152	152	152	150	145	55
P3HB–PU	152	152	152	152	152	152	150	145	50
C1	150	150	150	150	150	150	148	145	50
C2	148	147	147	146	146	146	144	141	50

**Table 2:** Summary of the rotational speeds used during extrusion using a twin-screw extruder, along with the monitored pressure level and the recorded load.

Material	Rotational Speed [rpm]	Pressure [bar]	System Load [%]
P3HB	130	26	72
P3HB–PU	130	28	83
C1	130	13	58
C2	130	10	51

**Table 3:** Technological parameters of injection molding of samples for testing the physical and mechanical properties of the P3HB+PU composition with urea additive.

Material	Plasticizing Pressure [bar]	Injection Pressure [bar]	Holding Pressure [bar]	Holding Time [s]	Injection Temperature [°C]	Cooling Time [s]	Mold Temperature [°C]
P3HB	190	600	400	25	165	25	30
P3HB–PU	200	550	350	26	176	25	40
C1	50	200	180	24	167	24	40
C2	50	200	180	24	167	24	40

**Table 4:** Mechanical properties of the tested materials obtained from uniaxial tensile tests: neat P3HB and its compositions with 10 wt.% PU (P3HB–PU) and with 10 wt.% PU and 1 wt.% urea (C1).

Sample	E_t_ [MPa]	σ_M_ [MPa]	ε_M_ [%]	σ_B_ [MPa]	ε_B_ [%]
P3HB	4926 ± 27	33.5 ± 1.4	1.19 ± 0.05	33.5 ± 1.4	1.42 ± 0.05
P3HB–PU	2361 ± 48	31.1 ± 0.5	4.28 ± 0.24	28.0 ± 0.8	4.22 ± 0.21
C1	3037 ± 46	28.1 ± 0.7	1.33 ± 0.08	28.1 ± 0.7	1.31 ± 0.08

**Table 5:** Mechanical properties of the tested materials based on the static three-point bending test: neat P3HB and its compositions with 10 wt.% PU (P3HB–PU) and with 10 wt.% PU and 1 wt.% urea (C1).

Sample	E_f_ [MPa]	σ_fC_ [MPa]	σ_fM_ [MPa]	ε_fM_ [%]	σ_fB_ [MPa]	ε_fB_ [%]
P3HB	4513 ± 35	-	69 ± 1.2	2.14 ± 0.15	69 ± 1.2	2.14 ± 0.15
P3HB–PU	2321 ± 36	47.1 ± 0.2	50 ± 1.3	4.62 ± 0.40	50 ± 1.3	4.62 ± 0.40
C1	3054 ± 54	-	39 ± 3.1	1.47 ± 0.23	39 ± 3.1	1.47 ± 0.23

## Data Availability

The original contributions presented in this study are included in the article material. Further inquiries can be directed to the corresponding authors.
